# Deep Semantics Analysis for Scale Development: Replicating Psychometrics Validation Process with Large Language Models

**DOI:** 10.3390/bs16081338

**Published:** 2026-08-04

**Authors:** Nicola Milano, Rosa Pizzo, Cristiano Scandurra, Maria Francesca Freda, Davide Marocco

**Affiliations:** Department of Humanistic Studies, University of Naples Federico II, Via Porta di Massa 1, 80125 Naples, Italydavide.marocco@unina.it (D.M.)

**Keywords:** construct validity, semantic–psychometric convergence, natural language processing, scale development, Academic Psychological Distress

## Abstract

The present study examines whether large language model (LLM) embeddings can approximate the psychometric validation process of a newly developed psychological scale. Using the Academic Psychological Distress Scale (APDS) as a case study, we replicated the full validation pipeline, traditionally performed on human response data, by applying exploratory and confirmatory factor analyses (EFA and CFA) to semantic similarity matrices derived from LLM embeddings. We first computed cosine similarity among all 58 APDS items and found a moderately strong correspondence with the participants’ correlation matrix (ρ = 0.57). An EFA performed on the embedding-based similarity matrix yielded a six-factor solution explaining 79.1% of the variance. The best-performing model (32 items, loading cutoff = 0.70) demonstrated acceptable model fit when tested on participants’ responses (CFI = 0.88; TLI = 0.87; RMSEA = 0.07–0.08; SRMR = 0.06) numerically comparable to the fit obtained in the original human-based validation based on 367 participants’ response. Reliability estimates were satisfactory for most factors (ω/α = 0.82–0.95), although one factor showed lower internal consistency. The percentage of overlap between human-derived and embedding-derived structures, indicated an overall structural correspondence of 64%, with factor-level overlap ranging from 50% to 100%. While embeddings reproduced most dimensions of academic distress, they failed to recover the demoralization factor and instead introduced an additional behavioral cluster. These findings suggest that LLM embeddings can capture substantial aspects of the latent structure of psychological constructs and can support early-stage, low-data, embedding-first scale development. We propose a linguistic validity pipeline as a methodological framework for integrating semantic embeddings into psychometric validation procedures, highlighting their promise as a complementary tool for human-centered assessment.

## 1. Introduction

The validation of psychological scales is a cornerstone of robust psychometric research, ensuring that instruments accurately and reliably measure the constructs they are designed to assess. Traditionally, this process relies on the analysis of human-generated responses to scale items, a resource-intensive and often time-consuming endeavor. The advent of large language models (LLMs) and sophisticated embedding techniques (Brown et al., 2020; Radford et al., 2019; Vaswani et al., 2017) presents a compelling opportunity to explore an alternative, data-driven approach. These models, trained on vast corpora of text, generate high-dimensional vector representations, also called embeddings, that encode semantic and contextual relationships, essentially capturing the meaning of words and sentences in a numerical format.

The field of self-report language-based psychological assessment has rapidly embraced the use of LLMs. Particularly, due to the great number of tests and items freely available, and to the possible impact for a general audience, the domain of personality assessment provides a suitable benchmark to introduce new approaches. Within a relatively short span, numerous studies have emerged (Abdurahman et al., 2024; Hitsuwari et al., 2024; Kjell et al., 2022; Nilsson et al., 2024), highlighting the potential of LLMs in this domain. Research has shown that LLM-derived embeddings surpass traditional approaches, such as bag-of-words, in predicting responses to personality items (Abdurahman et al., 2024). Moreover, recent studies have demonstrated how LLMs can effectively cluster semantically similar items under their hypothesized constructs, achieving alignment with human-derived factorial structures without relying on response data (Guenole et al., 2025; Hussain et al., 2024; Milano et al., 2025a; Wulff & Mata, 2025). Despite extensive evidence that these methods can reproduce the factorial structures observed in human responses, they have almost exclusively been applied to already validated item pools and well-established tests from the classical literature. A critical reading of this literature reveals three recurring limitations. First, the evidence base rests almost entirely on already validated item pools, so it shows that embeddings can recover a factor structure that empirical validation has already confirmed, but not that they can anticipate one before any human data exist. Second, most studies apply a single, static embedding model and stop at the exploratory stage (e.g., PCA or EFA on a similarity matrix), leaving the confirmatory and reliability stages of validation, and their robustness, largely untested. Third, recent work cautions that the apparent psychometric signal in item embeddings may be driven by explicit lexical cues rather than by the underlying construct: when the tell-tale wording of an item is set aside, the coherence that established questionnaires appear to show can weaken substantially (W. Song et al., 2025). The question that remains unanswered is therefore not merely methodological but substantive: can the semantic structure of a scale, as encoded by a language model, predict the factorial validity that human responses will later reveal, at the earlies, pre-validation, stage of scale development, and thereby flag poorly correlated items before any data are collected? In this study, we investigate how these models perform at the very outset of the psychometric validation process, when all items are considered prior to the refinement and reduction typically driven by exploratory factor analysis. Specifically, we used a complete set of unvalidated items to address a fundamental question: Can the latent semantic structure of a psychological scale, as captured by a language model, align with the psychometric properties derived from human responses from the very beginning of the validation process, thereby enabling the identification of poorly correlated items and accelerating item refinement and exploratory factor analysis?

To investigate this, we apply LLM embeddings to the validation of the Academic Psychological Distress Scale (APDS; Scandurra et al., 2025). Academic distress has emerged as a critical indicator of student psychological functioning in academic contexts. However, prior research has been limited by the absence of a clear conceptual framework and the lack of a dedicated, multidimensional measurement tool. The APDS was developed to address this gap, offering a comprehensive instrument aligned with the refined construct of Academic Psychological Distress (APD). The APDS is a particularly suitable test case for the present purpose for three reasons. First, it is a newly developed instrument whose complete, pre-refinement pool of 58 hypothesised items is available, which lets us enter the pipeline at exactly the stage—before EFA-driven item reduction—that prior embedding studies could not access. Second, it is explicitly multidimensional, spanning affective, cognitive, somatic, and relational facets of academic distress (Scandurra et al., 2024; Scandurra et al., 2025), providing a non-trivial, multi-factor target against which semantic recovery can be tested. Third, a full human-based validation of the same items on independent samples has recently been published (Scandurra et al., 2025), giving a rigorous, like-for-like benchmark of factor structure, model fit, and reliability for direct comparison. This study therefore uses the APDS as a case study to explore the potential role of LLM embeddings in psychometric validation: unlike previous studies that relied on already validated item sets, we work with the full set of hypothesized items proposed in the original study (Scandurra et al., 2025), enabling us to replicate the entire validation process and highlight similarities and differences between human data and LLM-based analyses. Therefore, this study tests the capacity of LLMs to anticipate the factorial validity of a newly developed instrument.

We integrate traditional validation methods with semantic embeddings derived from a modern transformer-based LLM. Our central hypothesis is that the semantic relationships between scale items, as quantified by embedding-based similarity, will reflect the empirical relationships observed in human response data. By comparing factorial structure, model fit, and reliability across both traditional and embedding-based approaches, we aim to demonstrate the potential of LLMs to function as a complementary—and potentially transformative—tool in psychometric validation. Indeed, this study aims to explore the feasibility of integrating exploratory and confirmatory factor analyses (EFA and CFA) on similarity matrices derived from language model embeddings. By replicating the entire psychometric validation pipeline, from dimensionality exploration to model confirmation, using semantic data rather than human responses, we test whether language models can support a semantically driven, minimally data-reliant validation process: one that is a-priori in its factor structure specification, yet requires a constrained empirical step to calibrate the item-loading threshold.

More broadly, this approach defines what can be described as a linguistic validity pipeline: a structured procedure that integrates artificial intelligence, psychology, and research methodology. Within this framework, large language models act as semantic proxies for human reasoning, enabling each stage of the psychometric validation process to be conducted on linguistic representations of test items, offering a new pathway for accelerating and enhancing construct validation while maintaining theoretical rigor.

Furthermore, we introduce a novel quantitative metric: the percentage overlap between human-derived and embedding-derived factorial structures. It can be seen as an indicator of semantic–psychometric convergence. Indeed, this measure quantifies the proportion of factors and items that align across the two validation methods, offering an interpretable way to assess the extent to which LLM-based representations mirror human-based construct organization.

In sum, the present study pursues a single, focused objective: to test whether the factorial validity of a newly developed scale can be anticipated from the semantic structure of its items before human responses are analysed. We address three research questions:

RQ1. Does the item-level cosine-similarity structure derived from LLM embeddings correspond to the inter-item correlation structure obtained from human responses?
RQ2. Can exploratory and confirmatory factor analyses performed on the embedding-derived similarity matrix reproduce the factor structure, model fit, and reliability obtained from the human-based validation of the same items?
RQ3. Where do the human-derived and embedding-derived structures converge and diverge, and what does this reveal about the constructs (e.g., demoralization) that embeddings do or do not recover?

Accordingly, the study makes three contributions: (i) a first end-to-end replication of the EFA–CFA validation pipeline on semantic-similarity data rather than human responses; (ii) a quantitative index of semantic–psychometric convergence (the percentage overlap between human- and embedding-derived structures); and (iii) a proof of concept for an embedding-first, minimally data-reliant workflow for early-stage scale development. We stress at the outset that the semantic-structural evidence produced by this pipeline is intended as preliminary evidence to support—not replace—conventional construct validation; the broader conceptual and methodological implications are developed in the Section 4.

## 2. Methods

### 2.1. Development of the APDS Items

For a comprehensive account of the APDS survey construction and full methodological procedures, we refer to Scandurra et al. (2025); here, we provide only a summary. They generated an initial pool of 61 items based on a theoretical model of Academic Psychological Distress (APD), capturing affective, cognitive, somatic, and social dimensions. Items from existing scales (e.g., Depression Anxiety Stress Scales, General Anxiety Disorder Scale, Patient Health Questionnaire) were adapted to the university context. Then, two focus groups with clinicians (n = 6) and university students (n = 17) were conducted to refine the items, ensuring representativeness, relevance, and clarity. This process resulted in a final pool of 58 items. This complete 58-item pool—prior to any EFA-driven reduction—is precisely the input required for the present analyses: it lets us evaluate whether embeddings can approximate the validation outcome at the earliest stage of scale development, when a researcher has only candidate items and no response data. Conceptually, the APD framework (Scandurra et al., 2024) models academic distress as a multidimensional construct encompassing affective (e.g., academic anxiety, negative self-worth), cognitive and motivational (e.g., demoralization), somatic (e.g., stress-related bodily responses), and relational (e.g., academic loneliness) components; the 58 items were written to sample these hypothesised dimensions, providing a structured target against which embedding-derived clustering can be compared.

### 2.2. Participant Recruitment and Data Collection

The present work is a secondary, computational re-analysis of the data set collected and reported in the original APDS validation study (Scandurra et al., 2025); no new participants were recruited and no additional data were collected for this study. It is best characterised as a methodological validation study with a cross-sectional design, in which previously collected item-response data serve only as a benchmark for the embedding-derived structure. The recruitment, sampling, consent, and ethics procedures summarised below are therefore those of the original study.

They recruited two separate samples for the Exploratory Factor Analysis (EFA) and Confirmatory Factor Analysis (CFA). The EFA sample (N = 326) exceeded the recommended 5:1 item-to-participant ratio for the 58 items (Comrey & Lee, 1992; Costello & Osborne, 2005). This sample was primarily composed of women (67.7%, n = 210) with a mean age of 23.42 years (SD = 3.93). For the CFA, they recruited 367 participants, exceeding the 10:1 ratio for the 37 items retained after the EFA. This sample had a mean age of 19.96 years (SD = 0.87) and was predominantly female (60.8%, n = 223). Data were collected online via the Qualtrics platform from university students in Italy who were at least 18 years old. All procedures adhered to the EU General Data Protection Regulation and the Declaration of Helsinki and were approved by the University of Naples Federico II Ethics Committee. This ethics approval refers to the original data-collection study (Scandurra et al., 2025; ethics protocol no. 18/2024). Because the present study is a secondary analysis of fully anonymised existing responses, no separate approval or exemption was needed.

Regarding recruitment and eligibility, participants in the original study were university students in Italy, aged at least 18 years, recruited among all the students of the University of Naples Federico II. Participation was voluntary, and all respondents provided informed consent before completing the online questionnaire; no personally identifying information was retained. Full details of the eligibility criteria and recruitment procedure are reported in Scandurra et al. (2025).

In this study we focus only on sample two because instead of using participants’ data to perform the EFA, we use the embeddings of the items and then perform the CFA on sample two to compare to the result found in Scandurra et al. (2025).

### 2.3. Computational Embeddings

In computational linguistics and natural language processing (NLP), embeddings are a fundamental technique for representing linguistic units—such as words, phrases, and sentences—as dense numerical vectors in a high-dimensional space. These vector representations are designed to capture semantic, syntactic, and contextual relationships, thereby converting symbolic linguistic data into a sub-symbolic format that is amenable to computational processing. The values within these vectors are not random but are learned from large text corpora, enabling them to encapsulate the rich linguistic information required for machine learning models (Brown et al., 2020).

Early approaches to vector representation, such as Word2Vec (Mikolov et al., 2013), produced static word embeddings, assigning a single, fixed vector to each word. A significant limitation of this method is its inability to account for polysemy; the same vector is used for a word regardless of its specific meaning in a given context. The advent of transformer-based models (Vaswani et al., 2017) revolutionized this paradigm by introducing contextual embeddings. Models like BERT (Devlin et al., 2019) and GPT (Conneau et al., 2017) generate embeddings that are dynamically adjusted based on the surrounding words in a sentence, yielding semantically richer representations of both words and entire sentences (Liu et al., 2019).

In this study, we employed embeddings derived from Sentence-BERT (SBERT) (Reimers & Gurevych, 2020), a specialized class of BERT-based models optimized for generating semantically meaningful sentence embeddings. SBERT is fine-tuned from a pre-trained BERT model, which itself is trained on extensive corpora like the English Wikipedia and Google Books, using tasks such as masked language modeling. SBERT utilizes a Siamese Neural Network architecture, which is trained on pairs or triplets of sentences. This structure modifies the embeddings to minimize the distance between semantically similar sentences in the vector space while maximizing the distance between dissimilar ones.

We specifically utilized Microsoft’s MPNet (Masked and Permuted Pre-trained Network) and its multilingual variant (K. Song et al., 2020), both members of the BERT family. MPNet is designed to produce high-quality embeddings for a range of NLP tasks. Given that our dataset and validators were Italian, we translated the 58 APDS items from Italian into English and evaluated them using the English-language MPNet model. Each item was first forward-translated from Italian into English by a bilingual translator; the English version was then independently back-translated into Italian by a second bilingual translator; finally, an expert compared the original and back-translated Italian versions. We note that any residual semantic shift introduced by translation is a limitation we return to in the Section 4. This decision was based on the premise that English models, trained on significantly larger corpora, typically outperform their multilingual counterparts, a known discrepancy in the field (Li et al., 2024; Wu & Dredze, 2020). It should be noted, however, that this cross-lingual approach introduces an inherent asymmetry: the cosine similarity matrix is derived from item embeddings computed in English semantic space, whereas the human Pearson correlation matrix reflects the responses of Italian university students. Psychologically nuanced constructs—particularly those with strong cultural or experiential grounding, such as demoralization—may be susceptible to semantic shift during translation, and this limitation is discussed further in the Section 4.

### 2.4. Similarity Measurement: Cosine Similarity

To analyze the underlying factor structure of the embeddings, we needed to quantify the relationships between them. For this purpose, we used cosine similarity, a common metric in semantic analysis. Cosine similarity measures the cosine of the angle between two non-zero vectors in an inner product space, providing a measure of their orientation irrespective of their magnitude. This metric is particularly useful in positive spaces, where the result is bounded between 0 and 1, with a value closer to 1 indicating higher semantic similarity. Formally,

(1) $$\cos\left(x,y\right)=\frac{\sum_{i=1}^{D}x_{i}y_{i}}{\sqrt{\sum_{i=1}^{D}x_{i}^{2}}\sqrt{\sum_{i=1}^{D}y_{i}^{2}}}$$

where **x** and **y** are two embeddings vector in a D-dimensional embedding space.

Equation (1) is very similar to classic Pearson correlation coefficient:

(2) $$\hat{r}=\frac{\sum_{i=1}^{D}\left(x_{i}-\bar{x}\right)\left(y_{i}-\bar{y}\right)}{\sqrt{\sum_{i=1}^{D}{\left(x_{i}-\bar{x}\right)}^{2}}\sqrt{\sum_{i=1}^{D}{\left(y_{i}-\bar{y}\right)}^{2}}}$$

When the mean of the embedding vectors is zero, cosine similarity is mathematically equivalent to Pearson correlation. This occurs because, with a zero mean, the centered vectors used for Pearson correlation are identical to the original vectors used for cosine similarity. As noted by Zhelezniak et al. (2019), embeddings generated by many modern language models tend to have means very close to zero, causing the two metrics to converge in practice. Given this convergence, and our preliminary analysis confirming it, we chose to use cosine similarity as our primary measure of correlation.

Our approach to analyzing the underlying structure of the embeddings follows a three-step process: (i) we generate embeddings for each test item; (ii) we compute the cosine similarity for every pair of embedded items, yielding a cosine similarity correlation matrix; and (iii) we apply exploratory factor analysis (EFA) to this matrix. Once the factorial structure has been extracted, we assess its fit by conducting a confirmatory factor analysis (CFA) on participants’ responses. We then compare the construct validity and reliability of our results with those obtained through the classical validation process reported by Scandurra et al. (2025). Finally, similarity between the human-based scale and the embedding-derived scale was calculated through the overlap percentage of the embedding-derived structure with respect to the one obtained from human data, both for each individual factor and for the overall scale.

Regarding software and reproducibility, item embeddings were generated in Python 3.10 with the sentence-transformers library using the pretrained all-mpnet-base-v2 model). Cosine-similarity computation, exploratory factor analysis, confirmatory factor analysis, and reliability estimation were performed in R 4.5.3 using the psych package 2.5.6 (EFA and ω/α) and the lavaan 0.7.2 package (CFA). Because embeddings are a deterministic function of the item text, the semantic input contains no missing data by construction. For the confirmatory analyses on participant responses there were no missing data because responses were mandatory.

## 3. Results

In this section, we present the results of the validation process for the psychometric test on the APDS, using semantic embeddings from LLMs. First, we compare the correlation matrices of the items, based on participants’ responses and on the cosine similarity derived from LLMs. Next, we extract the factorial structure from the LLMs and test it using CFA on the respondents’ data. Finally, we assess the degree of overlap between the structures obtained from participants and those derived from the LLMs.

### 3.1. Correlation Between Items

**Figure 1:**
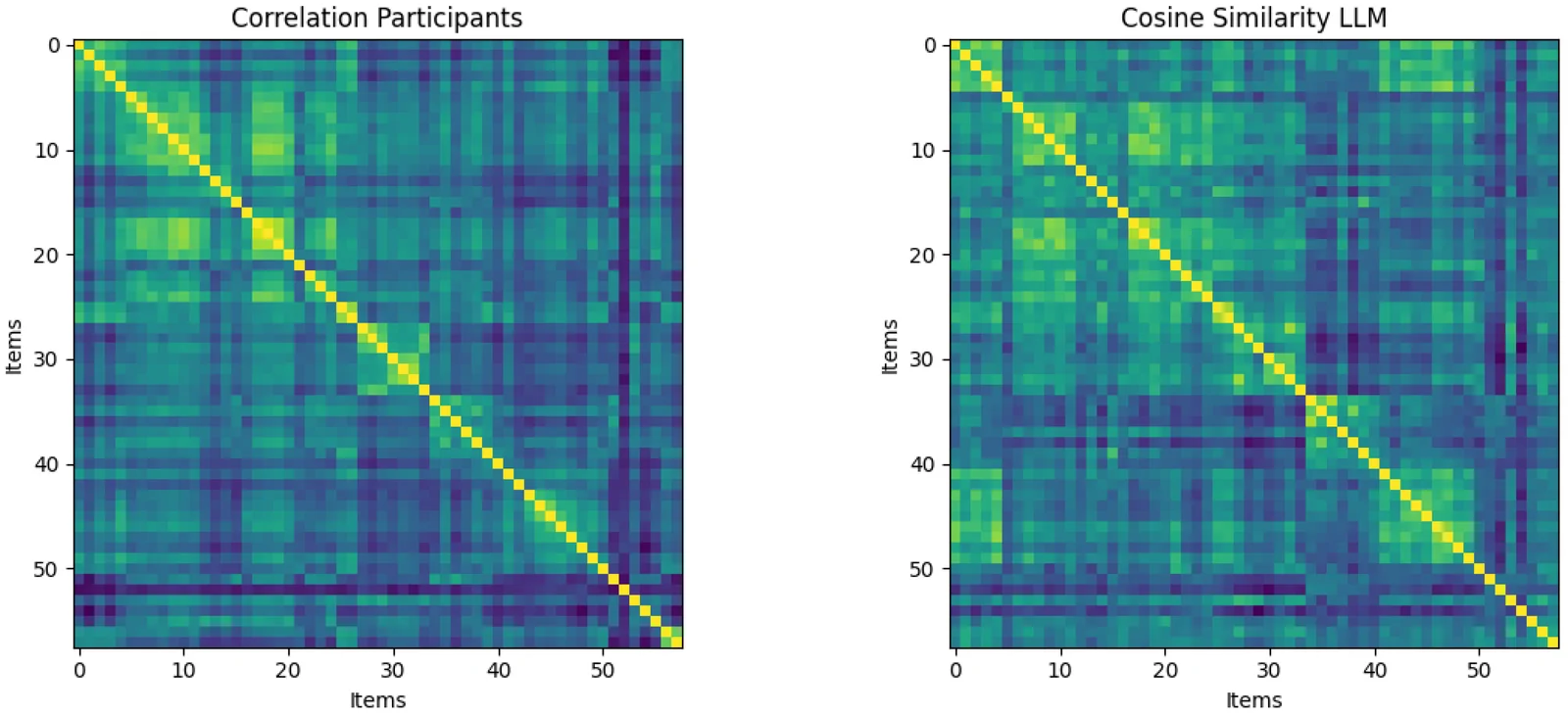
Cosine Similarities Matrix for the Items’ Embedding Obtained with the LLMs (**right**) and Correlation Matrix of the Participants’ Response (**left**). Dark areas correspond to cosine similarity 0, yellow areas correspond to cosine similarity equal to 1.

For each of the 58 items, the corresponding embedding vector was retrieved as described in the Section 2, and a 58 × 58 cosine similarity matrix was computed. Figure 1 presents a comparison between the Pearson correlation matrix of the items, derived from participants’ responses, and the cosine similarity matrix obtained from the language model embeddings. The figure illustrates that the correlation patterns observed in the participant data are, to a considerable extent, already reflected in the cosine similarities of the embeddings.

**Figure 2:**
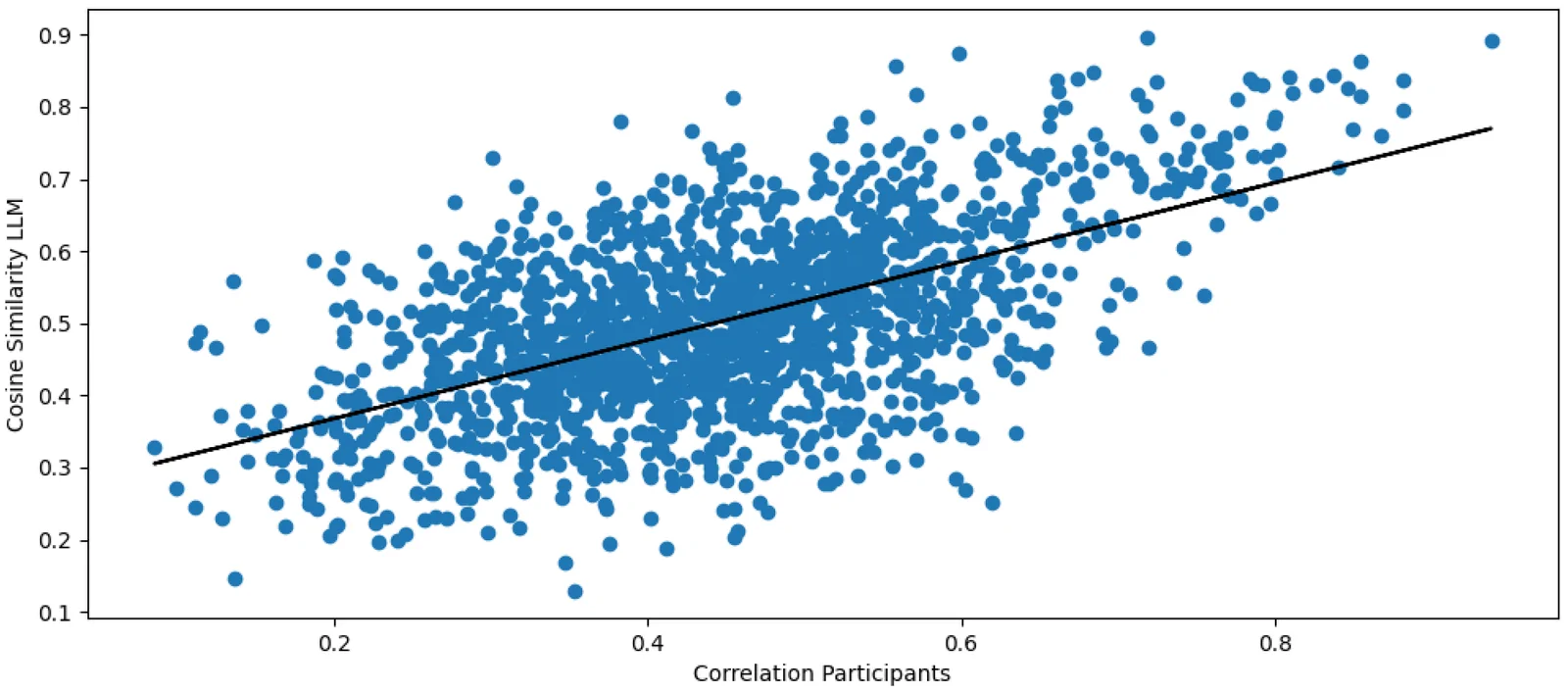
Spearman Correlation between the Upper Triangles of the Cosine Similarity Matrix of the Embeddings and Correlation Matrix of the Participants’ Responses (N = 1682).

To provide a quantitative assessment, we calculated the Spearman correlation between the upper triangles of the two matrices, obtaining a coefficient of 0.57 (Figure 2). Spearman’s ρ was chosen because the paired similarity and correlation values are bounded and non-normally distributed and are expected to relate monotonically rather than strictly linearly. Because the cells of a similarity matrix are not mutually independent—each item contributes to many cells—the significance of this association was evaluated with a permutation test (1000 permutations) rather than a parametric test that assumes independent observations; the correspondence was statistically significant (p<0.01). This result indicates a moderately strong correspondence between the two measures. Building on this evidence, we conducted an exploratory factor analysis on the cosine similarity matrix of the embeddings to identify the underlying factorial structure.

### 3.2. EFA on LLMS Embeddings

Previous studies have applied exploratory factor analysis (EFA) or principal component analysis (PCA) directly to cosine similarity matrices, reporting higher correspondence with the factorial structures obtained from human respondents (e.g., Feraco & Toffalini, 2025; Guenole et al., 2025; Milano et al., 2025a). Following this approach, we conducted an EFA on the cosine similarity matrix with minimal residual method as factor extraction and oblimin rotation. Using the eigenvalue-greater-than-one criterion, we identified a six-component factorial structure explaining 79.1% of the total variance (Table 1).

To refine factor allocation, we tested several six-factor solutions by varying the threshold for item loadings. In the original validation study (Scandurra et al., 2025), a cut-off of 0.50 was employed. However, given that cosine similarities derived from embeddings tend to overestimate the relations observed in human data, we applied a stricter range of thresholds, from 0.50 to 0.70 in increments of 0.05. This range was motivated entirely by the known systematic upward bias of cosine similarity matrices relative to human response correlations—a theoretical consideration documented in the embedding literature—and not by exploration of the participant data itself. Each resulting structure was then tested using confirmatory factor analysis (CFA) on the responses of 367 participants. The best-performing solution was obtained with a cut-off of 0.70, yielding a structure with 32 items. It is important to note that this threshold-calibration step constitutes the sole point of contact between the embedding-derived structure and human response data: the factor structure itself—the number of factors and the assignment of items to factors—was determined entirely from the semantic similarity matrix, without reference to participant responses. The pipeline is therefore best characterised as a-priori in structure and empirically calibrated in threshold, rather than fully data-free.

The resulting factors corresponded closely to the hypothesized initial dimensions of the scale, with the following specification:

a = V7, V11, V18, V19, V20, V23, V24, V25

b = V28, V29, V31, V32, V33, V34

c = V35, V36, V37, V38, V39, V40

d = V1, V3, V4, V5, V42

e = V43, V47, V48, V49

f = V52, V55, V58

This model produced marginal fit indices—below the conventional CFI/TLI ≥ 0.90 threshold (Hu & Bentler, 1999)—($\chi^{2}$/df = 3.33; RMSEA = 0.07, 90% CI [0.07, 0.08]; CFI = 0.88; TLI = 0.87; SRMR = 0.06), numerically comparable to the CFA results obtained from the EFA based on participants’ responses (Scandurra et al., 2025: $\chi^{2}$/df = 3.24; RMSEA = 0.08, 90% CI [0.07, 0.08]; CFI = 0.88; TLI = 0.87; SRMR = 0.06). (The interpretation of this comparability is developed in the Section 4).

Furthermore, to estimate the variability between the structure derived from the language model and that obtained from participants’ data, we performed a resampling analysis. Specifically, we randomly drew 10 subgroups of 200 responses (with replacement, allowing for overlap across subgroups) from the total of 367 participants and applied CFA to each subgroup. For each approach, we computed the average RMSEA, SRMR, CFI, and TLI, along with their standard errors, thereby quantifying the stability and error margins of the language model–based versus participant-based structures (Table 2).

We also assessed the reliability coefficients for the 32 retained items across the sample. Overall, the factors demonstrated satisfactory psychometric properties, including adequate score variability and high internal consistency. Reliability was evaluated using Cronbach’s alpha and McDonald’s omega, with coefficients ranging from 0.82 to 0.95 except for factor 6 that exhibits a poor reliability with a coefficient of 0.6. Detailed results for each factor are reported in Table 3.

**Figure 3:**
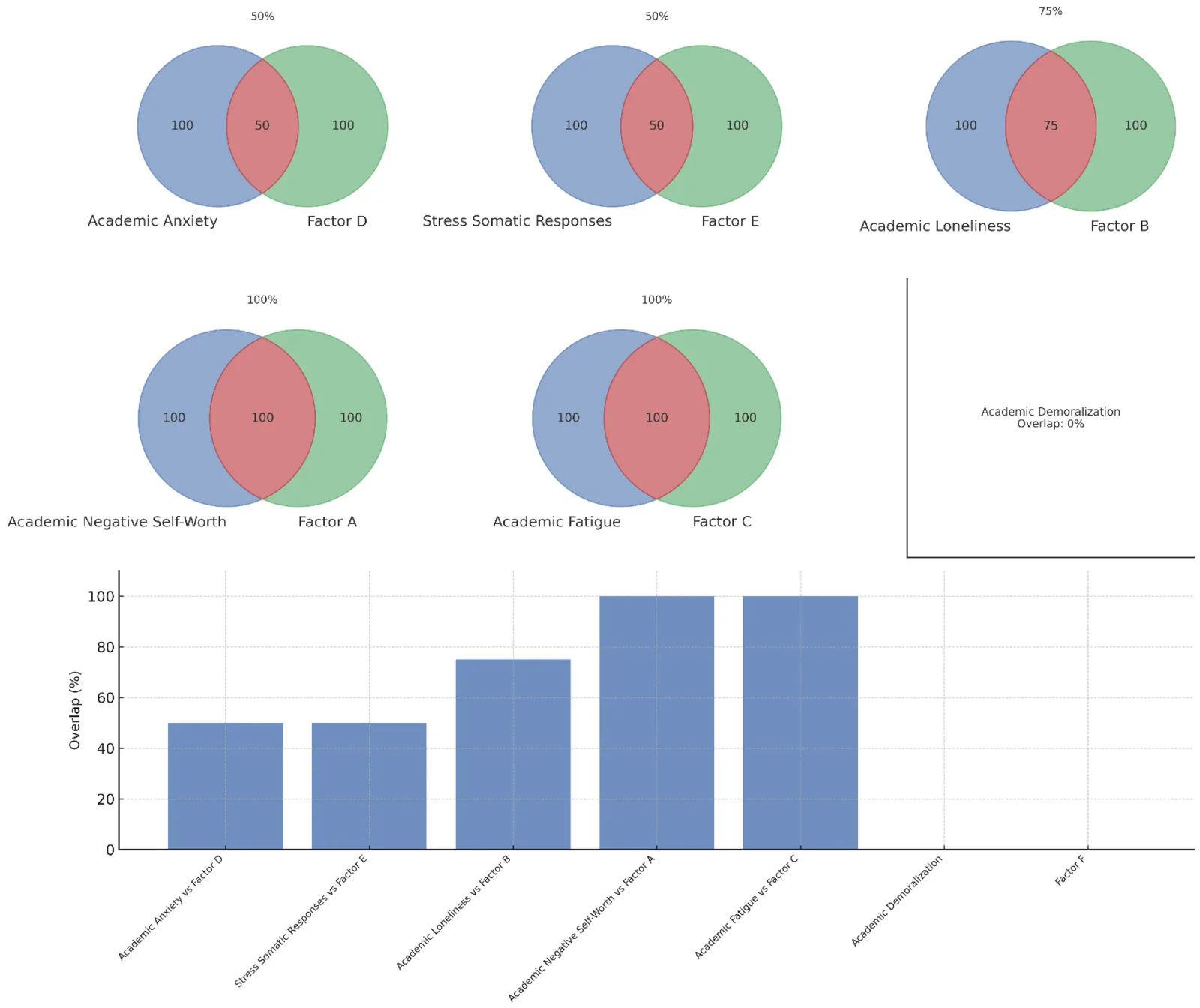
Overlap Percentages Between Human-Based and Embedding-Derived Factor Structures for Academic Psychological Distress Scale (APDS) Items.

Finally, regarding the similarity between the human-based scale and the embedding-derived scale, the results demonstrate overlap percentages for each individual factor ranging from 50% to 100%, with an overall overlap percentage of 64% (see Figure 3 and Table 4).

In particular, all factors are confirmed except for one (Demoralization), while an additional factor (Factor F) emerges in the embeddings.

## 4. Discussion

The present study explored whether sentence embeddings generated by transformer-based models could approximate the psychometric validation process of the APDS originally developed by Scandurra et al. (2025). Instead of relying solely on participants’ item-level responses, we analyzed the semantic similarity of test items as captured by large language models, specifically MPNet-derived embeddings, and examined their alignment with human data.

Moreover, a key methodological contribution of this study is the introduction of a quantitative index of semantic–psychometric convergence: the percentage overlap between the human-based and embedding-derived factorial structures. Unlike conventional fit indices, which assess model adequacy within a single analytic framework, the overlap index directly quantifies the structural correspondence across two distinct validation sources: human response data and language-model embeddings.

This metric reflects the proportion of items and factors that coincides across both approaches, providing an interpretable measure of how closely semantic similarity patterns replicate empirical factorial structures. Therefore, the percentage overlap can serve as a new indicator of semantic structural validity, complementing the traditional evidence of construct validity and supporting the broader integration of computational semantics into psychometric evaluation.

Our results showed a moderately strong correspondence between the correlation matrix derived from participants’ responses and that obtained from embeddings (ρ = 0.57). This finding suggests that contemporary embedding models capture latent semantic structures that overlap with human interpretations of item similarity, in line with prior evidence that embeddings encode psychological constructs at a lexical level. Importantly, the exploratory factor analysis of embedding-derived similarity revealed a six-factor structure that paralleled the original human-based structure, accounting for 79.1% of variance. This convergence supports the view that embeddings can serve as proxies for item-level relations in psychometric research.

To our knowledge, no previous research has attempted to apply both exploratory and confirmatory factor analyses to embedding-based similarity data. Methodologically, this work constitutes a first comprehensive replication of the traditional psychometric validation process, from exploratory to confirmatory stages, conducted entirely on semantic similarity data derived from large language model embeddings. Previous studies have primarily focused on exploratory analyses (e.g., PCA or EFA) applied to embedding-based matrices. In contrast, we demonstrate that embeddings can sustain the full validation cycle, including confirmatory factor analysis and reliability estimation, thereby providing a proof of concept for an embedding-first, minimally data-reliant psychometric workflow in which factor structure is entirely a-priori and only the loading threshold requires a constrained empirical calibration step. Compared to these prior efforts, our findings are broadly consistent with reports that embedding similarity predicts human factor structure (e.g., Feraco & Toffalini, 2025; Guenole et al., 2025; Milano et al., 2025a; Wulff & Mata, 2025), but they qualify that literature in two ways. First, because we entered the pipeline with an unvalidated pool and carried it through to CFA and reliability, we showed that the correspondence survives the more demanding confirmatory stage—not only the exploratory stage those studies examined—while also exposing where it fails (the demoralization factor). Second, whereas earlier work generally reports overall agreement metrics, our overlap index makes the partial nature of that agreement explicit (64% overall), which we regard as a more honest characterisation of what embeddings currently deliver than a single headline correlation.

However, the comparison between the two structures (human-based vs. embedding-derived) revealed that some items, which in the human-based validation were distributed across different factors, converged into a single cluster (Factor F) in the embedding-based models. Specifically, these items included procrastination (“I tended to procrastinate, that is, put off study commitments and/or exams.”), lying about exams (“I sometimes lied about my exams and the progress of my studies.”), and excessive sacrifice of free time (“I excessively sacrificed my free time because of worries related to University.”). These items share a behavioral, action-oriented framing rather than describing emotional states or cognitions. We label the resulting cluster academic maladaptive coping, but we emphasise that this label was assigned post hoc, after inspecting the items grouped by the embeddings, and should not be read as a latent dimension that the model discovered. Alternative accounts are at least as plausible. The three items may cohere simply because they share concrete behavioral verbs and self-referential “I”-statements that embeddings weight heavily, rather than because they index a single coping construct. They are also not obviously unidimensional in content: procrastinating and lying about academic progress plausibly reflect avoidance, whereas excessively sacrificing free time may instead reflect over-engagement. Consistent with a cluster driven by surface lexical similarity rather than a coherent behavioral construct, this factor also showed the weakest reliability (see below). We therefore present Factor F as a tentative, semantically induced grouping that would require targeted item writing and empirical testing before it could be interpreted as a psychometric dimension.

By contrast, one factor identified in the human-based scale (i.e., demoralization) did not emerge in the embedding-based models. One possible explanation is that, the demoralization items, although expressing the same psychological condition (loss of hope, lack of meaning, and absence of pleasure), employ heterogeneous linguistic formulations: some refer to the future (“I do not expect anything good from my academic future”), others to meaning (“I felt that studying was pointless”), and still others to pleasure (“I felt that there was nothing about my university experience that brought me pleasure or excitement”). We caution, however, against concluding from this single case that LLMs are by default unable to represent demoralization. The non-recovery of this factor is open to several competing explanations that our design cannot fully disentangle: (i) heterogeneous wording—the demoralization items refer variously to the future, to meaning, and to pleasure, so they may be semantically dispersed even though they index one construct; (ii) translation effects—demoralization is culturally grounded and may lose specificity when Italian items are rendered in English and embedded in English semantic space; (iii) insufficient semantic specificity of these particular items relative to neighbouring constructs such as anxiety and negative self-worth; and (iv) limitations of the specific embedding model used here, since a single static sentence encoder need not be representative of LLMs in general. This pattern is also consistent with recent evidence that the structure LLMs appear to show on questionnaire items can be driven by explicit lexical cues rather than by the target construct (W. Song et al., 2025). We therefore treat the missing demoralization factor as an open question rather than as evidence of a general representational limit, while noting that it may partly reflect the embodied, subjective character of demoralization (Chersoni et al., 2021). This underscores the importance of integrating computational and human-based analyses, rather than treating the former as a substitute or alternative.

Confirmatory factor analysis further reinforced this correspondence. The best-performing embedding-based six-factor model (32 items, cutoff = 0.70) achieved fit indices that, while marginal under conventional psychometric standards (CFI/TLI ≥ 0.90; Hu & Bentler, 1999), are numerically comparable with those reported by Scandurra et al. (2025) from participant data (CFI = 0.88, TLI = 0.87, RMSEA = 0.07–0.08). This parallel is particularly meaningful given that the embedding-derived model is strictly a-priori: the number of factors and the assignment of every item to a factor were determined from semantic similarity data alone, without consulting modification indices, freeing cross-loadings, or dropping items based on participant responses. Under these far more demanding conditions, achieving the same degree of fit as a fully data-driven pipeline—confirmed by resampling analyses showing indistinguishable mean CFI (LLM: 0.865 ± 0.013; participants: 0.853 ± 0.015) and TLI (LLM: 0.847 ± 0.015; participants: 0.836 ± 0.017) across subsamples (Table 2)—constitutes a meaningful proof of concept for the embedding-first approach. Reliability estimates for embedding-derived factors were also satisfactory (α/ω = 0.83–0.95), closely mirroring the psychometric robustness of the human-validated APDS except for one factor that appears to be not reliable and more generated from semantic cluster of words rather from an underlying effective behavior. Taken together, these findings highlight the potential of embedding-based methods to approximate, and in some cases replicate, traditional validation outcomes.

Nevertheless, some discrepancies emerged. Embeddings tended to overestimate inter-item similarity, requiring stricter thresholds for factor retention. This suggests that language models capture broader semantic overlap than humans perceive, potentially conflating conceptually adjacent but psychologically distinct items. Moreover, while embeddings reproduced the broad factorial architecture of the APDS, they cannot replace the lived experiences that inform human responses, especially in domains such as emotional salience or context-specific interpretation. Thus, embeddings should be regarded as complementary to, rather than substitutes for, human data in validation studies (Milano et al., 2025b). Two additional limitations deserve explicit acknowledgment. First, the cross-lingual design of the present study—in which Italian items were translated into English for embedding computation, while the human correlation matrix was derived from Italian respondents—introduces an inherent semantic asymmetry. Translating psychologically nuanced items inevitably incurs some degree of semantic shift, and culturally grounded constructs such as demoralization may be particularly vulnerable to such loss. The failure of the embedding-based pipeline to recover the demoralization factor may therefore reflect, at least in part, the attenuation of culturally specific meaning during translation into English, rather than a fundamental limitation of embeddings per se. Future work should assess whether Italian-language or multilingual embedding models yield different results, and more generally should ensure linguistic and cultural alignment between the embedding model and the target population. Second, although the factor structure in the present study is strictly a-priori, the selection of the final loading threshold (λ = 0.70) was guided by CFA fit on participant data, constituting a constrained data-dependent step. The pipeline is therefore more accurately characterised as embedding-first and minimally data-reliant rather than fully data-free. Future research should investigate whether this threshold generalises across item pools and embedding models, or whether a theoretically derived correction factor for the known upward bias of cosine similarity matrices could eliminate the need for empirical calibration altogether. Third, the evidence reported here rests on a single scale, a single psychological domain (academic distress), a single language pair (Italian–English), and a single embedding architecture, so claims of broad applicability must remain provisional. Academic-distress items are also lexically repetitive—many share explicit references to “University”, “studying”, “exams”, and student identity—which almost certainly makes semantic clustering easier here than it would be for scales with subtler, more heterogeneous, or more metaphorical wording. The method is therefore likely to be most useful for constructs whose items carry clear, content-laden lexical markers and behaviorally concrete phrasing, and least reliable for constructs that are abstract, affectively subtle, culturally idiosyncratic, or deliberately worded to avoid obvious keywords. Establishing where on this spectrum the approach breaks down will require replication across multiple scales, domains, languages, and embedding models.

Theoretically, our findings raise important implications for psychometrics. Embeddings encode statistical regularities of language shaped by vast corpora, and therefore reflect socially and culturally shared meanings. This allows them to approximate structural validity at the semantic level. However, psychometric constructs such as distress are not purely semantic: they are grounded in subjective experience and behavior. Future work should therefore consider hybrid approaches, in which embeddings guide or constrain item development and early-stage validation, but are systematically cross-validated against participant data.

To prevent the over-interpretation of embedding-derived structures, it is worth stating explicitly what the method is, and is not, intended to decide. We see embeddings as most valuable at the item-pool refinement stage, before data collection, where they can support four concrete tasks: (i) flagging near-duplicate or redundant items through very high pairwise similarity; (ii) detecting poorly worded, off-construct, or semantically isolated items that cluster with no others; (iii) proposing provisional domains to organize a large item pool; and (iv) prioritizing ambiguous items for expert review. Decisions that must remain the domain of expert judgement and participant data include the final assignment of items to constructs, the confirmation of dimensionality, the interpretation of factors, and all evidence bearing on external, criterion, and predictive validity. In this division of labour the embeddings can help to triage and hypothesize, while humans and respondents confirm.

Looking further ahead, the linguistic validity pipeline need not remain tied to a single embedding model. Recent work on multi-agent LLM systems shows how distinct roles can be allocated across cooperating models and coordinated through shared memory and structured communication (Hang et al., 2026; Sultimov et al., 2026). Although these systems are not methodological precedents for embedding-based factor analysis, they suggest a natural extension of the present proposal—an AI-assisted pipeline in which separate agents are responsible for item generation, semantic evaluation, construct critique, and psychometric verification, cross-checking one another and flagging disagreements for human adjudication. Situating our single-model results within this broader trajectory clarifies that embeddings are one component of a potentially larger, more autonomous scale-development workflow rather than an end point.

Beyond the empirical findings, this study proposes the concept of a linguistic validity pipeline, that is, an integrated framework that bridges artificial intelligence, psychometrics, and research methodology. The pipeline encompasses a full sequence of validation steps performed on linguistic data: item embedding, semantic similarity estimation, exploratory and confirmatory factor analyses, and the assessment of structural convergence with human-based data.

In doing so, it operationalizes a new form of validity evidence that we can call semantic-structural validity, grounded in the internal coherence of item language rather than participant responses. We are careful to frame semantic–structural validity as a form of preliminary evidence about the internal, linguistic coherence of an item set, not as a substitute for, or an alternative form of, full construct validation. Semantic coherence reflects shared linguistic content, whereas psychometric validity concerns how a construct is experienced by respondents and how it relates to external variables. A semantically coherent cluster is therefore a hypothesis about structure to be tested against participant data, not the evidence of construct validity in itself.

Such an approach may serve as a methodological bridge between computational semantics and psychological measurement, providing a scalable and theoretically consistent pathway for early-stage validation.

## 5. Conclusions

This study provides an initial demonstration that sentence embeddings can reproduce, to a considerable extent, the psychometric structure of a validated psychological scale. By applying exploratory and confirmatory factor analyses to embedding-based similarity matrices, we found that large language models are capable of capturing latent factorial structures that align with human data, including satisfactory reliability and marginal—though comparable—model fit, achieved without any empirical tuning of the factor structure itself.

The integrated EFA–CFA pipeline applied to semantic similarity data represents a novel methodological advancement, demonstrating that large language models can reproduce not only the exploratory but also the confirmatory stages of psychometric validation.

However, embeddings should not be viewed as replacements for empirical validation with human samples. Instead, they may function as a powerful methodological supplement—supporting early item screening, reducing redundancy, and providing theoretical checks during the scale development process. Such applications could accelerate instrument design while preserving the necessity of human-centered testing for establishing construct validity and criterion-related evidence.

## Figures and Tables

**Table 1:** Percentage of Variance for Each Factor and Total Cumulative Variance Retrieved by the EFA on the Embeddings.

Factor	Eigenvalue	% of Variance	Cumulative %
A	18.73	18.70	18.7
B	12.92	16.88	35.6
C	5.43	12.69	48.3
D	2.74	12.54	60.8
E	1.66	11.48	72.3
F	1.36	6.81	79.1

**Table 2:** Mean and Standard Deviation of the CFI, TLI, RMSEA, and SRMR Indices across 10 Subsamples of 200 Responses Randomly Drawn from the Full Sample of 367 Participants.

Model	Index	Mean	Standard Deviation
Participants	CFI	0.853	0.015
	TLI	0.836	0.017
	RMSEA	0.105	0.004
	SRMR	0.076	0.003
LLM	CFI	0.865	0.013
	TLI	0.847	0.015
	RMSEA	0.106	0.003
	SRMR	0.086	0.009

**Table 3:** Cronbach’s Alpha and McDonald’s Omega Coefficients for Each Factor Derived from the Analysis of Item Embeddings.

	Mean (STD)	Cronbach’s Alpha	McDonald’s Omega
Factor A	2.25 (1.00)	0.90	0.90
Factor B	1.98 (1.14)	0.95	0.95
Factor C	1.36 (1.09)	0.92	0.92
Factor D	2.17 (0.87)	0.90	0.90
Factor E	1.30 (0.98)	0.81	0.82
Factor F	1.67 (0.68)	0.60	0.60
APDS total scale	1.82 (0.869)	0.97	0.97

**Table 4:** Comparison of Human-Based and Embedding-Derived Factor Structures for Academic Psychological Distress Scale (APDS) Items.

Items	Human-Based Factors	Embedding-Derived Factors
1. When I thought about University, I felt tense or nervous.	Academic Anxiety	Factor D
2. I had difficulty taking my mind off University.	*	*
3. Thinking about University made me restless and unable to stay calm.	*	*
4. I couldn’t stop worrying about University.	Academic Anxiety	Factor D
5. Thinking about University made me feel a sense of anguish, as if I were about to have a panic attack.	**	Factor D
6. I worried that I would fail in my university work.	*	*
7. I worried that I would be a disappointment as a student.	Academic Negative Self-Worth	Factor A
8. I felt sad when I thought about myself as a student.	*	*
9. I felt helpless when I thought about myself as a student.	*	*
10. I felt hopeless as a student.	*	*
11. I felt I was pretty worthless as a student.	Academic Negative Self-Worth	Factor A
12. I felt frustrated when I thought about myself as a student.	*	*
13. I couldn’t expect anything good from my academic future.	Academic Demoralization	***
14. I felt that studying was pointless.	Academic Demoralization	***
15. I felt that there was nothing about my university experience that brought me joy or excitement.	Academic Demoralization	***
16. I had no motivation to study.	*	*
17. University difficulties led me to have negative thoughts.	*	*
18. I felt I was failing as a student.	Academic Negative Self-Worth	Factor A
19. I felt that I was a disappointment as a student.		Factor A
20. I felt incompetent and inadequate as a student.	Academic Negative Self-Worth	Factor A
21. As a student, I felt trapped.	*	*
22. I felt angry toward University.	*	*
23. I felt angry at myself because I could do more as a student.	**	Factor A
24. I felt guilty for not doing everything a good student should do.	**	Factor A
25. I felt ashamed of being the student that I am.	Academic Negative Self-Worth	Factor A
26. I felt exhausted and emotionally drained by the university experience.	*	*
27. I felt overwhelmed by the university experience.	Academic Anxiety	***
28. I felt lonely and isolated at University.	Academic Loneliness	Factor B
29. I felt excluded at University.		Factor B
30. When I was at University, I felt out of place.	Academic Loneliness	***
31. I felt like there was no one I could turn to for help with my university problems.	Academic Loneliness	Factor B
32. I felt that no one could understand me regarding my university problems.	**	Factor B
33. I felt alone with my university problems.	**	Factor B
34. I felt inadequate in my relationships with others when I was at university.	Academic Loneliness	Factor B
35. I had difficulty concentrating while studying and/or during lectures.	Academic Fatigue	Factor C
36. I couldn’t think clearly while studying and/or taking an exam.	**	Factor C
37. I got distracted easily while studying and/or during lectures.	**	Factor C
38. I had great difficulty remembering the concepts I was studying.	Academic Fatigue	Factor C
39. My mind went blank while studying and/or taking an exam.	**	Factor C
40. When I tried to study, I felt tired and/or had no energy.	Academic Fatigue	Factor C
41. When I sat down to study, I got a headache.	*	*
42. Thinking about University made me sleep badly.	**	Factor D
43. Thinking about University made me lose and/or excessively increase my appetite.	**	Factor E
44. I had tremors and/or a continuous increase in sweating when I thought about university.	Academic Stress Somatic Responses	***
45. Thinking about University made my breathing irregular.	Academic Stress Somatic Responses	***
46. I had an accelerated and/or irregular heartbeat when I thought about University.	*	*
47. Thinking about University made my stomach feel empty and/or my throat feel lumpy.	Academic Stress Somatic Responses	Factor E
48. Thinking about University made me feel nauseous and/or vomit.	Academic Stress Somatic Responses	Factor E
49. I had abdominal pain and/or intestinal problems when I thought about University.	**	Factor E
50. When I thought about University, I cried or felt like crying.	*	*
51. I got easily angry with others because of University.	*	*
52. I tended to procrastinate, i.e., put off study commitments and/or exams.	Academic Fatigue	Factor F
53. I used substances (e.g., “smart drugs” or cognitive enhancers) to improve my academic performance.	*	*
54. I avoided lectures, peers, and university activities because taking part in university life made me feel uncomfortable.	*	*
55. I sometimes lied about my exams and the progress of my studies.	**	Factor F
56. Academic worries and/or difficulties made me seriously consider dropping out.	Academic Demoralization	***
57. Worrying about University kept me from living my social life the way I wanted to.	Academic Anxiety	***
58. I excessively sacrificed my free time because of worries related to University.	**	Factor F

* = Items that were not included in the final APDS structure after CFA, based on neither the Human-Based Analysis nor the LLMs; ** = Items excluded from the final APDS structure after CFA according to the Human-Based Analysis, but retained by the LLMs; *** = Items retained in the final APDS structure after CFA according to the Human-Based Analysis, but excluded by the LLMs.

## Data Availability

Data available upon request.
